# LACC1 regulates changes in the intestinal flora in a mouse model of inflammatory bowel disease

**DOI:** 10.1186/s12876-023-02971-5

**Published:** 2023-10-17

**Authors:** Zheng-Yuan Xu, Jin-Chun Wang

**Affiliations:** 1 Jiangsu Key Laboratory of Immunity and Metabolism, Xuzhou Medical University, Xuzhou, 221004 Jiangsu China; 2 Department of Gastroenterology, The Affiliated Shuyang Hospital of Xuzhou Medical University, No.9, Yingbin Avenue, Shuyang County, Suqian, 223600 Jiangsu China

**Keywords:** DSS, Inflammatory bowel disease, Intestinal flora inflammatory factor, LACC1

## Abstract

**Background:**

The aim of this study was to explore the mechanism whereby LACC1 regulates the intestinal flora in a mouse model of inflammatory bowel disease (IBD).

**Methods:**

C57BL/6 and *Lacc1*^*−/−*^ mice were used to establish a mouse model of IBD induced by dextran sodium sulfate (DSS). The effects of *Lacc1* deletion in mice were evaluated. Changes in the body weight and stool blood were recorded daily. After 7 days of successful modeling, the mice were sacrificed, blood was collected from the eyeballs, the entire colon was dissected and separated, and the length of the colon was measured.

**Results:**

Compared with the wild-type (WT) DSS model group, the *Lacc1*^*−/−*^ DSS model group showed a significantly higher disease activity index score (*P* < 0.05), significantly faster weight loss (*P* < 0.05), and a significantly shorter colon (*P* < 0.05), indicating that the colonic mucosal tissue was seriously damaged in the *Lacc1*^*−/−*^ DSS model group (*P* < 0.05). Serum IL-1β, IL-6, TNF-α, and IFN-γ levels were significantly higher in the *Lacc1*^*−/−*^ DSS model group than the WT DSS model group. Principal coordinate analysis showed that there were significant microbiome differences between the WT, *Lacc1*^*−/−*^, WT DSS model, and *Lacc1*^*−/−*^ DSS model groups (*P* < 0.05). Linear discriminant analysis effect size analysis showed that under natural conditions, *Lacc1*^*−/−*^ mice had significant changes in their intestinal flora compared with control mice (LDA value > 3 or < 3, *P* < 0.05).

**Conclusions:**

*Lacc1* deletion aggravates DSS-induced IBD in mice.

## Background

Inflammatory bowel disease (IBD) is a chronic disease that affects the digestive system. Crohn’s disease (CD) and ulcerative colitis (UC) are the main types of IBD [1]. In the past few decades, the incidences of UC and CD have shown upward trends, reaching their highest levels at the beginning of the 21st century [2]. In recent decades, the industrialization and urbanization of non-developed countries and regions, such as Asia, South America, and the Middle East, have led to the development of IBD in residents of these regions. As such, IBD has now become a global disease [3]. Epidemiological statistics in China show that the prevalence and incidence of IBD are increasing, and that IBD has become a common disease. IBD mainly affects the digestive tract, but it can also affect the joints, eyes, and other organs. These affects ultimately endanger human health and affect the quality of life of patients [1]. However, the etiology and pathogenesis of IBD remain unclear [4].

Research on the pathogenesis of IBD is limited. IBD was previously thought to be caused by genetic, environmental, and immune factors. However, an imbalance in the intestinal flora is common in patients with IBD and is mainly manifested by changes in diversity. The main manifestations are a decrease in intestinal flora diversity; a decrease in the abundance of beneficial bacteria producing short-chain fatty acids, represented by Firmicutes; and an increase in the abundance of adherent-invasive *Escherichia coli* (AIEC) [5, 6]. However, in patients with IBD, the lack of symbiotic bacteria causes AIEC and other pathogenic bacteria to become dominant in the intestinal tract [7]. Recent studies have found that microorganisms participate in the pathogenesis of IBD by interacting with the immune system [8]. Currently, it is believed that an imbalance between helper Th17 cells and regulatory T cells is an important cause of IBD onset [9]. Th17 cells are not only involved in the pathogenesis of IBD through the production of pro-inflammatory cytokines, but interleukin (IL)-17 produced by these cells can also stimulate colonic dendritic cells to produce IL-12 and IL-23, thus aggravating intestinal inflammation [10].

Laccase domain 1 (LACC1), also known as C13orf31 or FAMIN, is strongly activated by lipopolysaccharide (LPS) and poly I:C in mouse bone marrow-derived macrophages. The LACC1 protein is highly expressed in inflammatory macrophages and plays an important regulatory role in many inflammatory diseases, such as arthritis and microbial infections. However, the biochemical mechanism of action required for LACC1 function remains largely unknown. Recent studies have shown that its expression is induced by macrophage colony-stimulating factor in an AKT/mTOR-dependent manner during human monocyte macrophage differentiation. LACC1-deficient mouse models show an increased incidence of arthritis, psoriasis, T-cell metastatic colitis (*Rag2*^*−/−*^ background), and intestinal bacterial infection (*Citrobacter rodentium* and *Salmonella enterica* serovar Typhimurium). Mice with LACC1 deficiency show increased proinflammatory cytokine levels and poorer intestinal bacterial clearance, which inhibits their anti-inflammatory and antibacterial functions. However, the mechanisms by which this enzyme mediates its wide range of effects remain unclear.

Relevant studies have shown that LACC1 is necessary for the removal of intestinal pathogens and resident intestinal bacteria [11]. The loss of LACC1 increases the abundance of intestinal bacteria and aggravates intestinal damage [12]. Current research results indicate that LACC1 is necessary for PDK1-dependent bacterial uptake and NF-κB- and MAPK-dependent induction of reactive oxygen species, reactive nitrogen species, and autophagy pathways in macrophages, and negative feedback regulates the elimination of bacteria by these cells [13]. LACC1 is potentially involved in the pathogenesis of IBD; however, there is no relevant animal study to verify this. This study aimed to detect changes in the intestinal flora due to regulation by LACC1 in a mouse model of IBD and to analyze the expression of LACC1 in a mouse model of IBD through in vitro experiments. This study provides ideas for the exploration of the pathogenesis of IBD and the search for new treatments.

## Methods

### Experimental animals

C57BL/6 wild-type (WT) mice, (specific-pathogen-free [SPF] grade; 9–12 weeks, average 10.61 ± 0.17 weeks; weight 20–24 g, average 22.50 ± 0.19) g) and *Lacc1*^−/−^ mice (SPF grade; 9–12 weeks, average 10.61 ± 0.17 weeks; weight 20–24 g, average 22.50 ± 0.19 g) were provided by Nanjing University Institute of Model Animals. After breeding, the mice were screened, identified, and selected for further use. This study was approved by the Ethics Committee of The Affiliated Shuyang Hospital of Xuzhou Medical University, and was carried out in accordance with the prescribed procedures in the “Regulations on the Management of Laboratory Animals of the People’s Republic of China”, and conformed to all ethical requirements (IACUC approval number GR20220012). The study protocols also met the 3 H Animal Welfare Principle, which prescribes reducing the use of animals as much as possible when the experimental conditions are met, ensuring a comfortable experience for the animals, and reducing the pain experienced by the animals. This study was conducted in accordance with the ARRIVE guidelines (https://arriveguidelines.org).

### IBD model establishment

After the animals were randomly grouped according to their body weight, the aqueous solution in the animals’ water bottles was replaced with a 3% dextran sodium sulfate (DSS; Sigma-Aldrich, St. Louis, Missouri, USA) solution. The DSS solution was stored in the dark at 4 °C and was replaced with a fresh solution every 2 days (DSS was administered on day 1, and the DSS solution was changed on day 3 and day 5). The DSS solution was replaced with DSS-free water on day 9. After ingesting water for 14 days, the previous DSS administration steps were repeated 2–3 times. After modeling, the mice were continuously observed and their weight change and the presence of blood in the stool were recorded daily. The disease activity index (DAI) score was used to determine the status of the mice. The control group was given normal drinking water for 7 days, during which the weight change of the mice and the presence of blood in the stool were recorded daily.

### Grouping and specimen collection

The mice were divided into the following four groups: C57BL/6 control (WT), C57BL/6 DSS model (WT DSS), *Lacc1*^*−/−*^ mouse control (*Lacc1*^*−/−*^), and *Lacc1*^*−/−*^ DSS model (*Lacc1*^*−/−*^ DSS).

After 7 days, blood was collected from the eyeball and serum was isolated by centrifugation at 4,500 rpm for 10 min. The entire colon of each mouse was dissected and separated, and the length and weight of the colon were measured. The colonic contents were cleaned using a medical cotton swab and set aside.

### Determination of water content in mouse feces

Mice of different genotypes and aged 8–10 weeks were divided into groups and raised separately. The feces of each mouse were collected separately. To ensure the freshness of the feces, the samples were collected in a 1.5 mL Eppendorf tube as soon as they were discharged from the body to avoid drying. The weight of the fresh feces samples was recorded. All collected feces samples were placed in the same batch in an oven at 60 °C for approximately 2 h until they had completely dried, and the weight of the feces was recorded again. The proportion of water in the mouse feces was determined by two weight calculations.

### Hematoxylin and eosin staining and histological damage score

Part of the colon tissue was removed to clean the contents, and it was rolled up and fixed in 4% paraformaldehyde. After dehydration and clearing, the samples were embedded in wax, sectioned, dewaxed, and staining with hematoxylin and eosin (HE). The histological damage of the colon was scored by an experienced pathologist. Fifteen high-power fields (400 ×) were randomly selected for each section, and the average score was calculated. The scoring system was as follows: inflammation: 0 points for none, 1 point for mild, and 2 points for severe; No: 0 points for lesions, 1 point for submucosa, 2 points for muscular layer, and 3 points for serosal layer; damage to crypts: 0 points for basement, 1 point for 1/3 destruction, 2 points for basal 2/3 destruction, 3 points for complete epidermis only, and 4 points for the destruction of all crypts and epithelium; and lesion range: 1 point for 1–25%, 2 points for 26–50%, 3 points for 51–75%, and 4 points for 76–100%.

### Enzyme-linked immunosorbent assays

Part of the colon tissue was cut and placed in 500 µL of RIPA lysis buffer, vortexed to fully lyse, and centrifuged at 14,000 rpm for 5 min. The resulting supernatant was taken, and IL-1β, IL-6, tumor necrosis factor-α (TNF-α), interferon-γ (IFN-γ) enzyme-linked immunosorbent assay (ELISA) kits were used to determine their concentration in colon tissue, following the manufacturer’s instructions.

### *16 S rDNA* sequencing analysis

*16 S rDNA* sequencing was performed using a second-generation sequencing platform (Shanghai Meiji Biomedical Technology Co., Ltd., Shanghai, China). Operational taxonomic unit (out) analysis was performed using the Usearch software platform (version 7.0, http://drive5.com/uparse/). The Silva database was used for the analysis (Release l32, http://www.arb.silva.de).

Raw reads were obtained from the sequencer, low-quality base sequences were removed, and downstream analysis was performed. Through clustering, the sequences were divided into several groups based on their similarities, with each group comprised of an OTU. After comparison with the Silva database, a specific OTU classification table was obtained, and the representative sequences of the OTUs were selected. Downstream alpha and beta diversity analyses were also performed. Principal component analysis is a type of linear dimensionality reduction that transforms a group of potentially correlated variables into a group of linearly correlated variables through orthogonal transformation. It is used to identify the overall characteristics of different groups of bacteria. The main components were identified using the built-in prcomp function of R, and data visualization was performed using the factoextra package.

The differential flora were identified using linear discriminant analysis effect size (LEfSe) analysis (http://huGenhower.sph.harvard.edu/galaxy/root?tool_id=lefseupload) according to the taxonomic composition of the sample and different grouping conditions to perform linear discriminant analysis (LDA) to identify communities or species that had significantly different effects on the classification of samples.

### Statistical analysis

The main data were processed using SPSS 21.0 (IBM, Armonk, NY, USA) and R. An independent-samples Student’s t test was used to compare the two groups. Numerical results were expressed as the mean ± standard deviation (SD), and *P* < 0.05 was considered to indicate a statistically significant difference between the two groups.

## Results

### Effect of *Lacc1* deletion on inflammatory bowel disease symptoms in mice

**Fig. 1:**
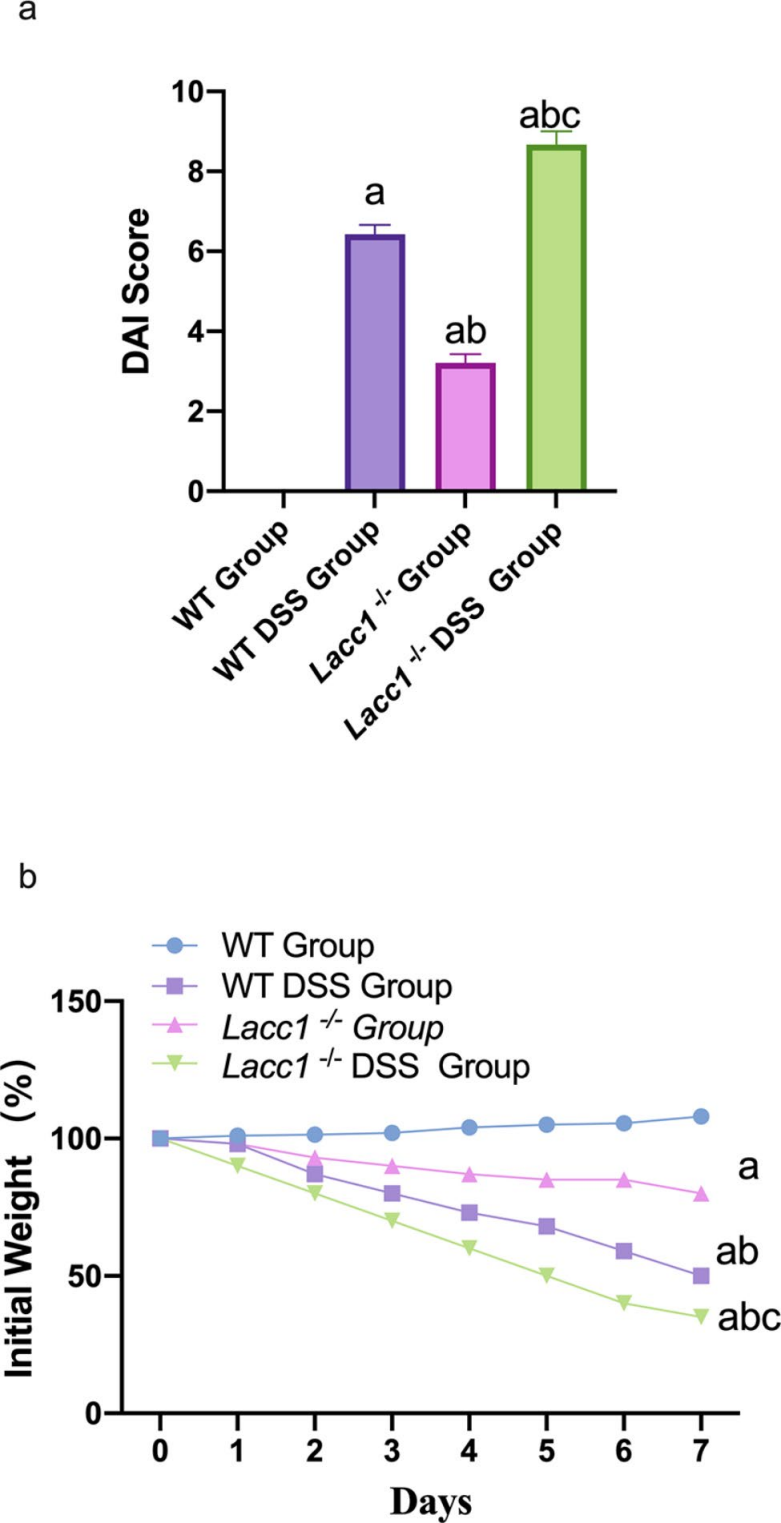
The effect of *Lacc1* deletion on the symptoms of inflammatory bowel disease in mice. **(a)** The disease activity index (DAI) score table was used to evaluate the severity of ulcerative colitis in mice, based on the symptoms, signs, and stool conditions. **(b)** Analysis of changes in the body weight of mice in each group. WT: wild-type mice; WT DSS: wild-type mice treated with dextran sodium sulfate (DSS); *Lacc1*^*−/−*^: *Lacc1*-deficient mice; *Lacc1*^*−/−*^ DSS: *Lacc1*-deficient mice treated with DSS. ^a^*P* < 0.05 compared with the WT group; ^b^*P* < 0.05 compared with the WT DSS group; ^c^*P* < 0.05 compared with the *Lacc1*^*−/−*^ group

A DAI score sheet was used to analyze the severity of UC in each group of mice based on symptoms, signs, and stool conditions. Our results showed that, compared with WT mice, *Lacc1*^*−/−*^ mice had a significantly higher DAI score after drinking 3% DSS (*P* < 0.05; Fig. 1a), reflecting the gradual aggravation of colitis. This indicated that Lacc1 has a protective effect on DSS-induced IBD in mice. In addition, our results showed that *Lacc1*^*−/−*^ mice had significantly more rapid weight loss than WT mice (*P* < 0.01, Fig. 1b), providing further evidence that Lacc1 has a protective effect on DSS-induced IBD.

### Effect of *Lacc1* deletion on intestinal injury in mice with inflammatory bowel disease

**Fig. 2:**
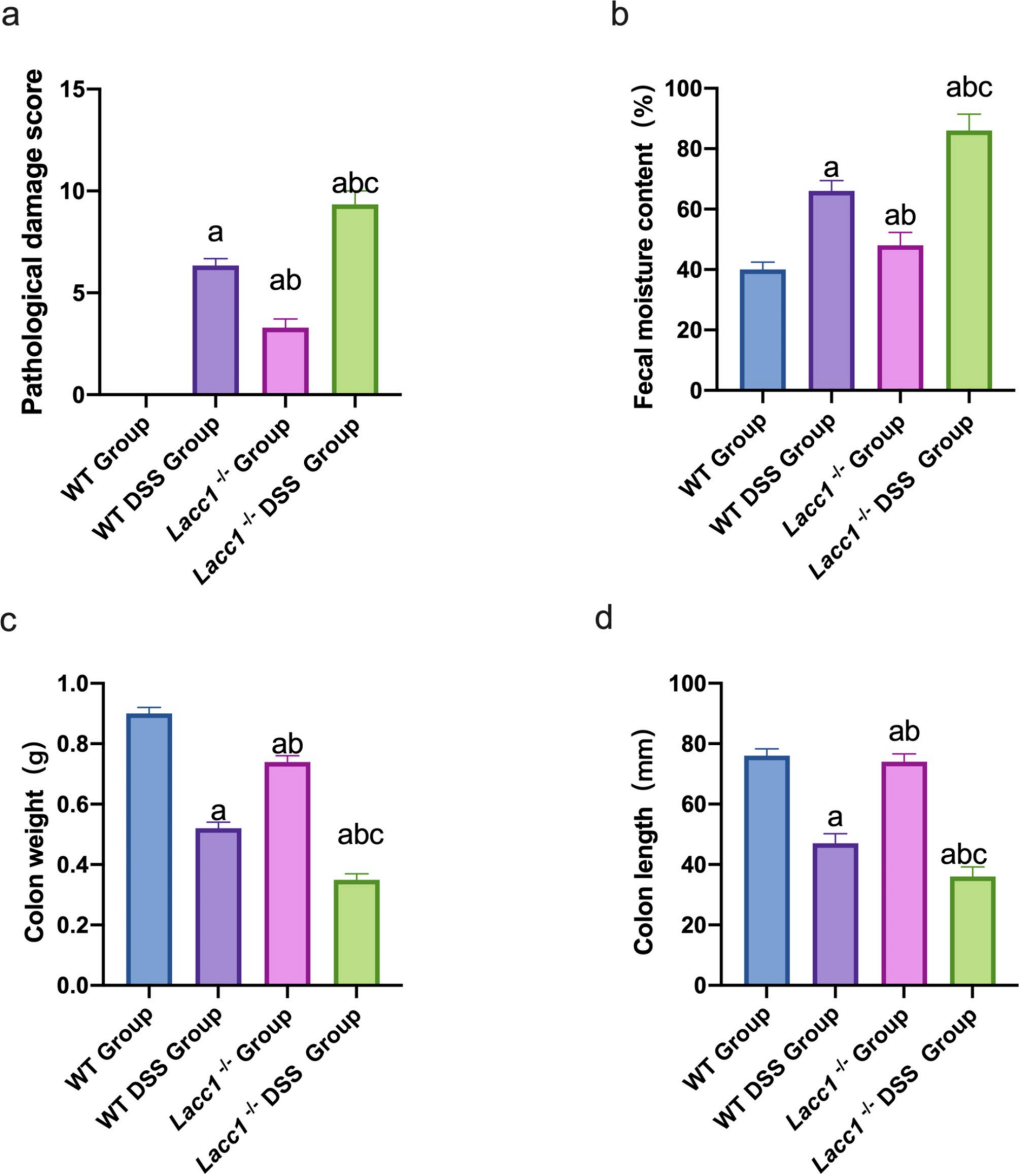
The effect of *Lacc1* deletion on intestinal injury in mice with inflammatory bowel disease. Comparison of **(a)** histopathological damage scores, **(b)** fecal water content, **(c)** colon length, and **(d)** colon quality of mice in each group. WT: wild-type mice; WT DSS: wild-type mice treated with DSS; *Lacc1*^*−/−*^: *Lacc1*-deficient mice; *Lacc1*^*−/−*^ DSS: *Lacc1*-deficient mice treated with DSS. ^a^*P* < 0.05 compared with the WT group; ^b^*P* < 0.05 compared with the WT DSS group; ^c^*P* < 0.05 compared with the *Lacc1*^*−/−*^ group

A histopathology scoring table was used to determine the degree of colonic mucosal tissue damage in each group. Colonic mucosal epithelial cells were absent in the model group, and most of the glands were incomplete. Inflammatory cells extensively infiltrated the body and typical inflammatory changes were seen. The *Lacc1*^*−/−*^ DSS group had a higher histopathological damage score than the WT DSS group (*P* < 0.05; Fig. 2a).

We measured the fecal water content of the mice in each group. Our results showed that fecal water content was significantly higher in the *Lacc1*^*−/−*^ DSS group than the WT DSS group (*P* < 0.05; Fig. 2b).

We extracted intestinal tissues from each group of mice to analyze intestinal inflammation. The large intestine was shorter in the *Lacc1*^*−/−*^ DSS group than the WT DSS group (*P* < 0.05; Fig. 2c). Moreover, edema and congestion of the colonic intestinal wall were visible to the naked eye, some symptoms of congestion appeared, and intestinal inflammation was obvious. The weight of the large intestine was significantly reduced in the *Lacc1*^*−/−*^ DSS group compared to the WT DSS group (*P* < 0.05; Fig. 2d).

### The effect of Lacc1 deletion on the intestinal inflammatory response in mice with inflammatory bowel disease

**Fig. 3:**
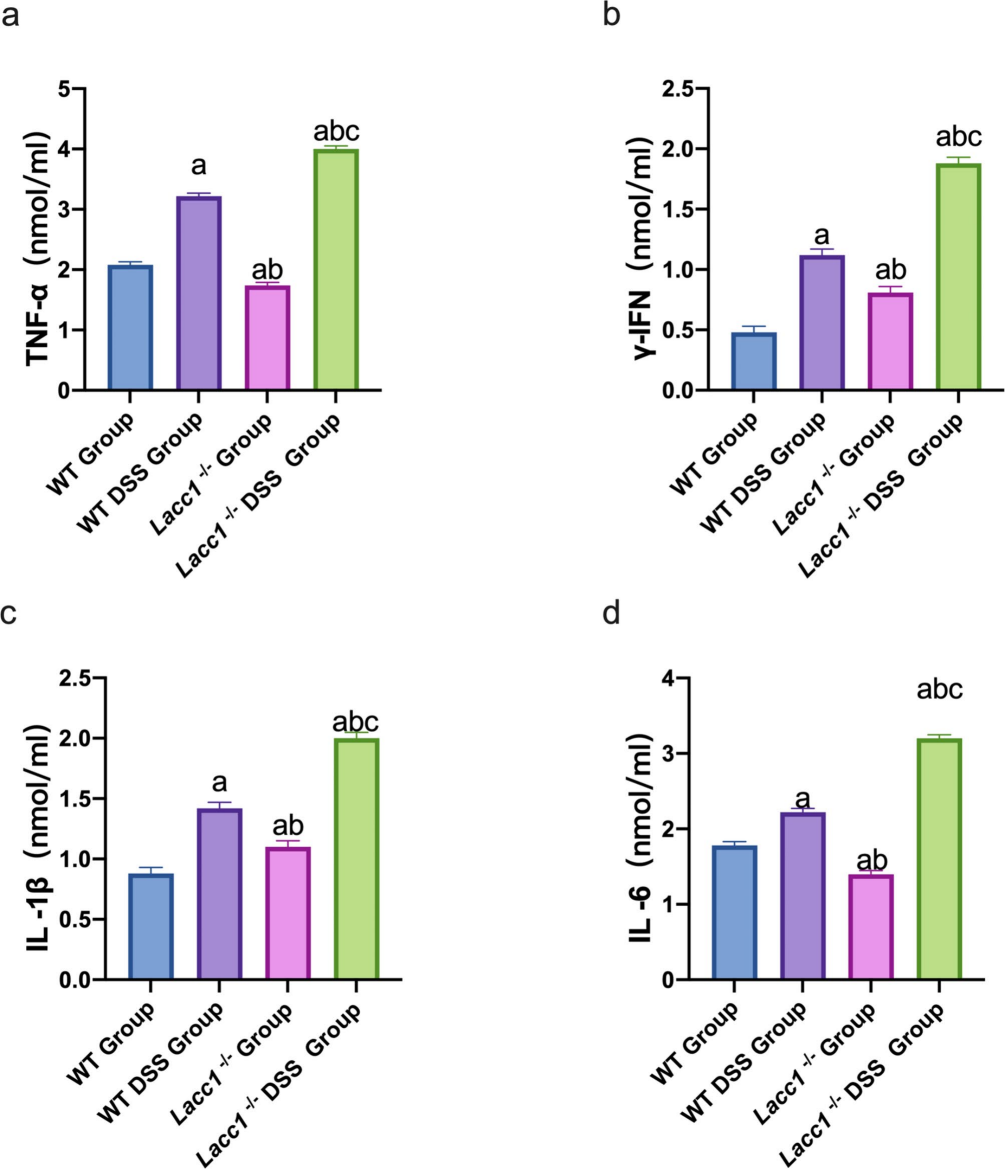
The effect of *Lacc1* deletion on the intestinal inflammatory response of mice with inflammatory bowel disease. Comparison of serum **(a)** IL-1β levels, **(b)** IL-6 levels, **(c)** TNF-α levels, and **(d)** IFN-γ levels of mice in each group. WT: wild-type mice; WT DSS: wild-type mice treated with DSS; *Lacc1*^*−/−*^: *Lacc1*-deficient mice; *Lacc1*^*−/−*^ DSS: *Lacc1*-deficient mice treated with DSS. ^a^*P* < 0.05 compared with the WT group; ^b^*P* < 0.05 compared with the WT DSS group; ^c^*P* < 0.05 compared with the *Lacc1*^*−/−*^ group

The occurrence and development of DSS-induced IBD is accompanied by the release of a large number of inflammatory factors. The expression levels of inflammatory factors in colon tissue can also reflect the state of inflammation of the colon. We used ELISAs to measure the levels of IL-1β, IL-6, TNF-α, and IFN-γ in the serum of WT and *Lacc1*^*−/−*^ mice. The results showed that the serum levels of the above-mentioned inflammatory factors were significantly higher in *Lacc1*^*−/−*^ mice than WT mice (*P* < 0.01, Fig. 3a–d), indicating that Lacc1 has protective effects against the inflammatory response.

### Effect of *Lacc1* deletion on gut microbes in mice with inflammatory bowel disease

Previous studies have found that changes in the homeostasis of the intestinal lumen environment can cause an imbalance in the intestinal flora in mice. We performed *16 S rDNA* analysis of the fecal flora of *Lacc1*^*−/−*^, WT, WT DSS, and *Lacc1*^*−/−*^ DSS mice to evaluate the intestinal bacteria of the mice.

### Group changes

**Fig. 4:**
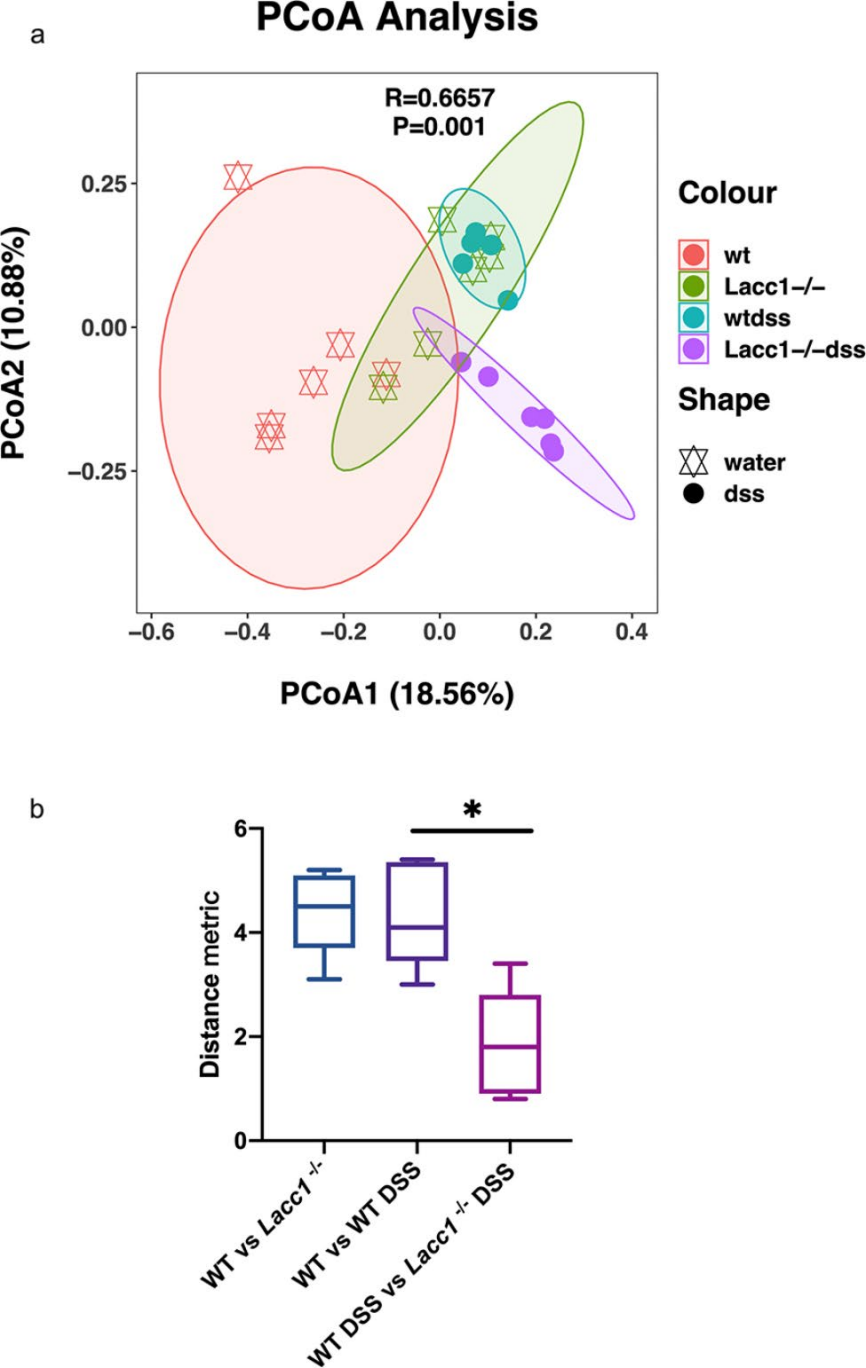
The effect of *Lacc1* deletion on the intestinal microbes of mice with inflammatory bowel disease. **(a)** As shown in the principal coordinate analysis diagram, differences in the overall abundance of microbial groups were observed between the groups of mice. **(b)** Beta diversity distance matrix analysis of mice in each group

Principal coordinate analysis showed that there were significant differences in the overall microbial groups among the WT, *Lacc1*^*−/−*^, WT DSS, and *Lacc1*^*−/−*^ DSS mice (*P* < 0.05, Fig. 4a). Furthermore, we compared the effects of differences in the intestinal flora of mice with different genotypes based on an analysis of the beta diversity distance matrix. There were statistically significant differences in the beta diversity distances of mice of different genotypes (*P* < 0.05, Fig. 4b). This showed that loss of *Lacc1* had a significant impact on the intestinal flora of mice with IBD.

### LEfSe analysis of mouse intestinal flora

LEfSe is widely used for intestinal microbial analyses. Multiple conditions, such as the biological classification of the intestinal flora, reveal changes in different groups of microbes. The non-parametric Kruskal-Wallis test was used to detect significant differences in the abundance of different taxa. Finally, LEfSe LDA was used to estimate the influence of the abundance of each species on the observed effects.

**Fig. 5:**
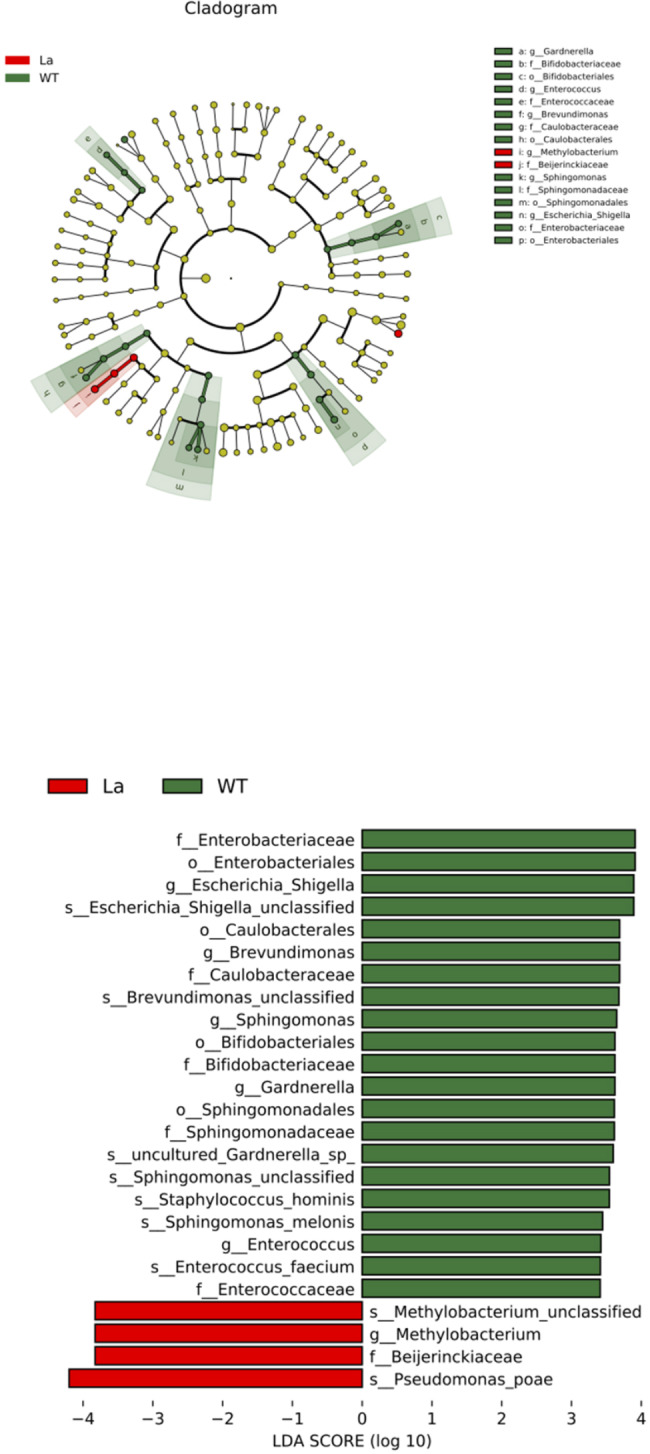
Linear discriminant analysis effect size analysis of mouse intestinal flora. **(a)** Evolutionary branch diagram; **(b)** distribution histogram

The *16 S rDNA* sequencing data for the intestinal flora of *Lacc1*^*−/−*^ mice were analyzed using LEfSe, and bacterial genera with significant differences between *Lacc1*^*−/−*^ and WT mice were identified. LEfSe analysis showed that, under natural conditions, *Lacc1*^*−/−*^ mice had significantly altered intestinal flora (LDA value > 3 or < 3, *P* < 0.05) compared with the WT group (Fig. 5a **and b**). The abundance of *Bacteroides uniformis* was significantly increased in the gut microbiota of mice in the *Lacc1*^*−/−*^ group. In addition, we found that *Lacc1*^*−/−*^ mice showed a significant upregulation of the abundance of multiple bacterial communities, including *Erysipelatoclostridium*, *Ruminococcus gnavus*_*group*, and *Escherichia*.

## Discussion

The intestinal flora is a key factor in maintaining human health. In this study, we described the changes in the intestinal tract, inflammatory response, and intestinal flora of *Lacc1*^*−/−*^ mice with IBD. We found that compared with WT mice, *Lacc1*^*−/−*^ mice with IBD had significantly worse intestinal damage; reduced colon weight; a shorter colon; elevated intestinal levels of inflammatory factors, such as IL-1β, IL-6, TNF-α, IFN-γ; and imbalanced intestinal flora. Therefore, we believe that Lacc1 plays a protective role in DSS-induced IBD in mice by reducing the inflammatory response and regulating the homeostasis of the intestinal flora.

IL-1β is an important cytokine involved in the pathogenesis of IBD [14–16]. After IL-1β binds to its receptor, it activates a variety of cytokines, including IL-6, through NF-KB [17]. Previous studies have shown that the expression levels of IL-1β and IL-6 are increased in mononuclear cells of the lamina propria of patients with IBD, and that these expression levels are closely related to inflammation and necrosis of the mucosa, the severity of disease, the extent of involvement, and recurrence [18]. TNF-α is one of the most important cytokines in the pathogenesis of IBD. It is secreted by inflammatory cells in the inflamed intestine and can cause further damage to the colon tissue. amplifies the intestinal inflammatory response and inhibits the synthesis and secretion of TNF-α. It effectively inhibits local inflammation in the intestinal tract [19]. Our findings showed that, compared to WT mice, *Lacc1*^−/−^ mice had an aggravated intestinal inflammatory response, indicating that Lacc1 may be involved in inhibiting the intestinal inflammatory response.

We found that *Lacc1*^*−/−*^ mice showed congenital spontaneous enteritis, similar to the clinical symptoms of patients with IBD. Therefore, we believe that Lacc1 plays an important protective role in maintaining homeostasis in the intestinal cavity and preventing the development of IBD. Recent research shows that changes in the environment of the intestinal cavity, due to conditions such as diarrhea or metabolic disorders, may cause an imbalance in the intestinal flora [20]. In this study, *16 S rDNA* sequencing showed that the intestinal flora of mice with IBD changed significantly in the absence of *Lacc1*. The abundance of *Bacteroides uniformis* was significantly increased in the intestinal flora of *Lacc1*^*−/−*^ mice.

*Bacteroides* is a genus of gram-negative bacteria comprising one of the two main bacterial groups in the human intestinal microbiome. Members of this genus are key bacteria in the human intestine that they degrade substances from the diet and ferment polysaccharides [21]. Previous studies have shown that *Bacteroides* are involved in the degradation of mucin, and an imbalance in abundance of the genus can destroy the mucosal barrier and allow potentially pathogenic microorganisms to further invade the host intestines, which is related to the development of IBD [22]. In addition, we also found that *Lacc1*^*−/−*^ mice had significant up-regulation of multiple types of flora, including *Erysipelatoclostridium*, *Ruminococcus_gnavus_group*, and *Escherichia*. It has been reported that *Erysipelas* is significantly more abundant in immune-mediated IBD. The upregulation of *Gastrococcus* and *Escherichia coli* are key factors that promote the occurrence and development of IBD. Numerous studies have shown that intestinal microbes significantly affect DNA damage, DNA methylation, chromatin structure, and noncoding RNA expression in intestinal epithelial cells [23, 24].

The main limitation of this study is that dysbiosis caused by *Lacc1* deficiency needs to be further confirmed. We will address this in future studies.

## Conclusions

In summary, we confirmed that *Lacc1* is a key gene in maintaining intestinal homeostasis, and we further revealed that the lack of *Lacc1* leads to an imbalance of the intestinal flora in mice. Understanding the mechanism responsible for the occurrence and development of IBD is useful for studying intestinal-flora-mediated pathogenesis, and will ultimately provide new strategies for the prevention, clinical diagnosis, and treatment of IBD.

## Data Availability

The datasets used and/or analyzed during the current study are available from the corresponding author on reasonable request.

## List of abbreviations

DSS: Dextran sodium sulfate
IBD: Inflammatory bowel disease
CD: Crohn’s disease
UC: Ulcerative colitis
Scfa: Short chain fatty acids
AIEC: Adhesion invasive Escherichia coli
Th17: Helper Th17 cells
Treg: Regulatory T cells
DC: Dendritic cells
LACC1: Laccase domain 1
LPS: Lipopolysaccharide
BMDM: Bone marrow derived macrophages
M-CSF: Macrophage colony stimulating factor
mLACC1: Mice with LACC1
WT group: C57BL/6 control group
WT DSS group: C57BL/6 DSS model group
*Lacc1*^*−/−*^ group: *Lacc1*^*−/−*^ mouse control group
*Lacc1*^*−/−*^ DSS group: *Lacc1*^*−/−*^ DSS model Group
TNF-α: Tumor necrosis factor-α
IFN-γ: Interferon-γ
OTU: Operational Taxonomic Unit
LDA: Linear discriminant analysis
SD: Standard deviation
